# Improvement of community health worker counseling skills through early childhood development (ECD) videos, supervision and mentorship: A mixed methods pre-post evaluation from Tanzania

**DOI:** 10.1371/journal.pgph.0001152

**Published:** 2023-06-05

**Authors:** Josephine Pascal Ferla, Michelle M. Gill, Teopista Komba, Amina Abubakar, Pieter Remes, Ola Jahanpour, Martha Mariki, Mary A. Mang’enya, Roland Van de Ven, Gretchen Antelman

**Affiliations:** 1 Elizabeth Glaser Pediatric AIDS Foundation, Dar es Salaam, Tanzania; 2 Elizabeth Glaser Pediatric AIDS Foundation, Washington, DC, United States of America; 3 Institute for Human Development, Aga Khan University, Nairobi, Kenya; 4 Development Media International, Mwanza, Tanzania; 5 President’s Office Regional and Local Government, Dodoma, Tanzania; 6 Community Development, Gender, Elderly and Children, Tanzania Ministry of Health, Dodoma, Tanzania; Institute of Tropical Medicine: Instituut voor Tropische Geneeskunde, BELGIUM

## Abstract

**Background:**

Community health workers (CHWs) play significant roles in improving health practices in under- resourced communities. This study evaluated an early childhood development (ECD) project in Tanzania assessing the effect of mobile video use, supervision and mentorship to improve quality of CHW counseling skills.

**Methods:**

CHWs participating in the Malezi Project in Tabora Region were enrolled in a mixed methods pre-post evaluation. CHWs previously trained in UNICEF’s Care for Child Development package were further trained in counselling caregivers on nurturing care and father engagement using videos. Health providers were trained to provide ECD-focused supervision/mentorship of CHWs in facilities and during home visits. At baseline and endline, CHWs completed interviews and trained study staff observed and scored CHW counseling sessions using a structured checklist which were reduced into six dimensions through principal component analysis: *introduce*, *educate*, *ask*, *plan/problem solve*, *interact/encourage*, and *responsive care*. Twenty-five in-depth interviews were completed with caregivers and four focus group discussions with CHWs were conducted.

**Results:**

Almost all (n = 107; 95%) 119 enrolled CHWs completed the expected eight observations (n = 471 baseline; n = 453 endline). At endline, more CHWs reported having one-on-one meetings with their supervisors (51% increasing to 75%; p < .0002) and that supervisors accompanied them to households for mentoring (60% increasing to 89%; p < .0001). We observed a shift in CHW counselling skills in clinic and home sessions. Scores in the categories of *introduce*, *plan/problem solve*, and *interact/encourage* significantly improved between baseline and endline; scores for *ask* and *educate* remained unchanged or decreased at both timepoints. Two-thirds of caregivers interviewed reported that father’s involvement with their child increased due to CHW visits. Male participation increased in home observation sessions from 5.6% at baseline to 17.6% at endline (p < .0001).

**Conclusion:**

Use of videos, supervision, and mentorship were associated with CHW performance improvements in providing nurturing care counselling and in father engagement, especially in home settings.

## Introduction

CHWs are recognized as an important cadre to expand access and utilization of health services in hard-to-reach rural areas [1–3]. Evidence supports the contribution community health workers (CHWs) can make to improved health outcomes, especially in low- and middle-income countries [1,2], such as reducing maternal mortality, reducing postnatal depression, and improving child growth [4] or mitigating effects of malaria, and promoting community-integrated management of childhood illnesses [5].

Studies are emerging on the use of community-based approaches in the provision of early childhood development (ECD)/nurturing care services integrated into routine health services. These studies emphasize the significance of integrating ECD services into existing national programs. For example, the integration of responsive care with nutrition programming using Lady Health Workers in Pakistan has proven to be a cost-effective intervention having measurable benefits in child development outcomes [6,7]. Another example of integrating ECD services into community-based platforms is from a study in Zambia focused on caregiver behaviors and parenting skills [8]. The Community Health Project (Jamii ni Afya) in Zanzibar noted the potential of scaling integrated interventions through national Community Health Volunteers using a mobile application to deliver maternal, newborn, child health, and child development services [9].

There is also evidence on the use of mobile health technology (mHealth) to improve health service provision through CHWs [1–3,10,11]. These mHealth, or digital, approaches include the use of tablets, cell phones, and smart phones [12], and sending SMS messages, video clips, and pictures/images. In community-based interventions, mHealth approaches have been employed to support health education, health promotion, decision making, data reporting, diagnosis, monitoring performance, and to receive reminders and alerts [1–3]. Several studies have reported on the use of mHealth tools to support CHW performance and provision of quality health care [5]. In India, short educational videos were shown by CHWs at home visits to support their ability to address issues related to HIV, alcohol abuse, nutrition, and breastfeeding [12,13].

Supervision and mentorship of CHWs are considered to be essential motivational factors which provide job satisfaction, retention [14], and performance improvement [15], particularly in settings where CHWs often have only primary education and limited opportunities for in-service training in public health [3,16]. Supervision, which ideally incorporates hands-on approaches to monitor outputs through record reviews and observation of patient interactions [15], often varies in intensity and fidelity to prescribed supervision guidelines. Mentorship involves continuous professional development provided by a mentor through frequent meetings and field-based collaborations to support more complex skills development, such as counseling, and provides opportunities for both mentors and mentees to develop positive professional relationships [16]. However, high-quality supervision and mentorship initiatives face many constraints in primary health care systems due to factors such as geographical scope and limited financial and human resources [1].

The Malezi Project in Tanzania included short educational videos on ECD used as job aids to support CHW performance in delivering consistent, high-quality counselling on ECD and nurturing care practices, along with supervision and mentorship of CHW practices. We aimed to assess the effectiveness of these interventions on CHW counseling skills and performance.

## Methods

### Project description and context

The Malezi Project, which translates to caring for young children in Swahili, was introduced in January 2018 in Nzega, Igunga and Tabora Municipal districts of the Tabora Region of Tanzania. The region is centrally located and among the largest in the country with a population of about 2.3 million residents; being among regions performing at 34.5% of basic needs poverty and 11.1% of Food poverty. The population is predominantly rural (87%) with agriculture as the major economic activity [17]. The project goal was to ensure that all children under three years achieve their developmental milestones.

The project targeted pregnant women and caregivers of young children at 86 health facilities and surrounding communities reaching a total of 82,545 families in 266 villages. It provided support to local government authorities and to health facilities in the three districts by integrating a responsive caregiving and early learning intervention into reproductive and child health and HIV services using the adapted UNICEF Care for Child Development (CCD) package [18]. CHWs were provided with Government-registered tablets, that were stored and charged by CHWs at the health facilities when not in use and contained five short (5–6 minutes) ECD video job aids. Videos were produced in the Swahili language in Tabora by a non-governmental organization in Tanzania, Development Media International, with local caregivers and CHWs modeling ECD practices (Swahili videos with English subtitles can be viewed at https://www.developmentmedia.net/project/malezi-ii/). Each video focused on a different topic and had specific messages for caregivers based on their child’s age at birth-6 months, 6–12 months, 12–24 months, and 2–3 years. A fifth video depicted expected developmental milestones from birth to three years of age. The videos were shown during counselling sessions to caregivers at their homes and in facilities during routine reproductive and child health service visits. The CHWs were trained to select a specific video according to the child’s age group and use it to discuss and demonstrate caregiver practices.

The project targeted all CHWs (188 women, 117 men) working with children under five years and their parents in supported health facilities. These CHWs had been recruited by the health facilities (prior to the project) to work with enhancing health, education and counseling services for mothers of young children attending the health facility. The project conducted a five-day training for CHWs using the CCD package and strengthened supervision and mentoring of CHWs during facility- and home-based sessions with caregivers. The training focused on building CHWs’ counseling skills and understanding ECD concepts. Specific topics provided in the training included how to counsel caregivers in identifying children’s cues and appropriately responding to them, providing a safe and stimulating environment in the home, understanding if their children are developing well, engaging in age-appropriate play and communication activities, identifying children with disabilities or developmental delays, and providing and following-up on referrals for specialized care, as needed.

CHWs were also trained for two days on how to facilitate group counseling and on using the educational videos as a working tool. The videos provided standardized messaging on the training topics described above, showing caregivers playing out different scenarios to illustrate the messaging for different age groups from birth to three years old. The CHWs were trained to use the videos in their counseling to generate discussion and opportunities for the caregiver to practice such behaviors with their child. CHWs also used memory cards to share ECD videos with caregivers who could view them on their own phones.

The project was managed by Elizabeth Glaser Pediatric AIDS Foundation (EGPAF) as NGO working in collaboration with the Ministry of Health. The project worked with the Regional and Council Health Management Teams (R/CHMTs), to provide mentorship and supportive supervision to CHWs. Members of the R/CHMTs team were capacitated to become trainers themselves in ECD, and field supervisors to the CHWs. A checklist was developed and used to supervise CHW performance on a quarterly basis. Supervisors and CHW met quarterly for experience-sharing and to practice counselling skills. CHW received monthly incentives for their work and supervisors received transport reimbursement when observing CHW during home counseling sessions.

### Study design and data collection

This study is a mixed methods pre-post evaluation of a sample of CHWs who participated in the Malezi Project in two (Nzega and Igunga) of the three project districts. Sample size calculations based on an expected 15% change (80% power) in CHW quality of counseling score from baseline to endline yielded a sample size of 90–123 CHWs, depending on different baseline scores (30%, 50%, 70%). Using the roster of existing CHWs, we purposively selected 17 health facilities out of 68facilities in the two districts to reach this approximate target. All CHWs (n = 120) from the 17 facilities were recruited into the study; 119 consented to join the study and completed a baseline interview (October 2019-March 2020) and 113 (95%) completed an endline interview (December 2020-March 2021). Five were withdrawn from the study during the intervention (two moved, two withdrew for health reasons, and one died), and one could not be traced at endline. Socio-demographic characteristics were collected at baseline, and we assessed CHW training, work effort and approaches, characteristics of CHW visits, use of the ECD videos, support from supervisors, and CHW’s satisfaction with their work at both baseline and endline.

An observation checklist, used for CHW supervision within the project and CHW evaluation scoring, was adapted from UNICEF’s CCD evidence-based counselling tool, but has not been validated in other settings. Adaptations to the tool were informed by the gaps identified in implementation, especially regarding CHWs’ counselling and interpersonal skills. In addition, changes were made to better reflect responsive caregiving support. Each CHW was assigned a median of five (interquartile range [IQR] 4,7) caregivers to visit monthly during the intervention and intervention fidelity monitoring data were collected monthly to document completion of CHW home visits to assigned caregivers.

Male and female study staff, aged 25–40 years, who were native Swahili speakers with previous research experience and residents of Tabora region, were trained on the protocol and study-specific procedures. They observed and scored 924 CHW counseling sessions (n = 471 at baseline; n = 453 at endline) using a structured evaluation checklist, with the goal of assessing two home and two clinic sessions for each CHW in each round. We included 891 (96%) in our pre-post analysis of CHW quality of counseling (n = 442 at baseline; n = 449 at endline), after excluding five that were incompletely scored (>6 missing items) and 28 missing endline data due to CHW withdrawal from the study. During the intervention (March 2020-January 2021), an additional 992 home (n = 661) and clinic (n = 331) sessions were observed and scored as part of the study’s fidelity monitoring. Psychometric analyses of the 23 common items assessed by the observation checklist tools for home and clinic sessions included baseline, process, and endline observations (n = 1883).

The primary outcome variables were defined in both continuous and binary form and were derived from the sum of scores of the 23 items common to the home/clinic observation tools. Raw scores, on a scale of 0–23, were calculated separately for home and clinic sessions at baseline and endline. Items with “yes (done)” or “no (not done)” response options were scored as “1” or “0” points, and items with “well done,” “partly”, or “not done” responses were scored as “1,” “0.5”, or “0” points, respectively. Raw scores were then standardized to a 0–1 scale. Scores for each of six dimensions are shown only in the standardized format to adjust for the varying number of items in each dimension score. The binary outcome variable is defined as a session score >16, where the cutoff of 16 represents the top tertile for all endline observation scores, combined across home/clinic. The distribution of CHWs according to this cutoff is determined by the mean CHW scores for clinic and home sessions at baseline and endline.

The six dimensions of the observation tool were: introduce where CHWs explained to caregivers the reason for the session; educate where CHWs suggested age-appropriate play or communication activities and explained their importance; ask where CHW asked caregivers questions on how they play or communicate with their children; plan/problem solve where the CHW probed on how caregivers planned to play and communicate with the child, and how they planned to involve the child’s father; interact/encourage included items related to whether the CHW praised the caregivers or allowed enough time for caregivers to practice play and communication with the child; and responsive care, referring to whether the CHW asked caregivers how they thought the child communicated needs or wants, and how they responded to those needs (Refer to full checklist tool in S1 Appendix).

Qualitative data were collected during the intervention period as part of a process evaluation in November/December 2020 by trained study staff. A total of 25 in-depth interviews (IDI) were completed with caregivers in their homes. Twenty-five caregivers with a recent CHW visit were purposively selected to participate in IDIs to ensure broad representation of experiences with their CHW counterparts, as nearly all caregivers interviewed were assigned to different CHWs. One additional caregiver each, who lived in the same area and had the same assigned CHW, was identified in case those on the initial list refused or were unavailable. Eleven caregivers needed to be replaced due to unavailability, the most common reason was due to farming demands during the rainy season. Four focus group discussions (FGD; two per district) were conducted, each with 6–8 CHWs (n = 29), in private rooms at hotels located in study communities. CHW participants were reimbursed for their time and transport to FGD locations. FGDs were conducted separately for male and female CHWs. CHWs were purposively selected to represent a mix of performance levels determined from observation scoring.

### Statistical analysis

We present frequencies, percentages, medians, and IQRs to describe CHW characteristics at baseline and endline, intervention fidelity, and classification of the CHWs according to their mean observation scores at baseline and endline. Statistical comparisons between groups were done using non-parametric tests (Wilcoxon rank sum test for continuous variables; McNemar’s or Chi-square test for categorical variables).

The observation tools for clinic and home sessions contained 23 core items common to both locations and both rounds of data collection. Two items were excluded from the final scale scores because they were almost universally done by all CHWs in all sessions (greeting and facing caregivers). Using the observation tool item scores as the unit of analysis, we conducted a principal components analysis to categorize the 23 items from the home/clinic observation tool into six dimensions ranging from 2–5 items each. All data management and statistical analyses were conducted using Stata (version 16; https://www.stata.com/).

IDI audio recordings were simultaneously transcribed verbatim and meaningfully translated into English transcripts that were used for analysis. Transcripts were then imported into a qualitative software program (Atlas V7.1). A draft codebook was developed with deductive codes based on questions from the interview guides. The codebook was revised to add inductive codes during the initial coding process. The coders were two experienced members of the study team trained on the protocol and in qualitative analysis. They coded the same transcripts twice, compared the assigned codes for similar text segments, added codes as needed, and resolved any discrepancies, resulting in the final codebook (S2 Appendix). The two coders then coded the remaining transcripts individually. Data were analyzed by other study staff using a thematic analysis approach by participant category (caregiver and CHW). This involved the careful reading of transcripts to identify recurrent patterns and themes and reducing data into matrices and text summaries by code. All of the analyses were reviewed and reduced further by one of the principal investigators. Quotes that reflected emerging patterns in the data, particularly when capturing sentiments in an eloquent or interesting way, were pulled from the transcripts and included in the summaries by code as part of the analysis. Multiple quotes were often identified to illustrate similar ideas; they were narrowed down during final write-up to those that most appropriately captured themes and were felt to be best presented in individuals’ own voices. This paper focuses on the qualitative results that help to contextualize the main quantitative findings. Major themes described below are the CHW role in planning and conducting home visits, use of video job aids to enhance ECD messaging and promote ECD behaviors, and home counseling sessions as an opportunity for greater father involvement. IDIs and FGDs explored additional topics such as general CHW support to caregivers, facility ECD services, and other proposed and existing platforms to promote ECD.

### Ethical considerations

The protocol for this evaluation was approved by the National Research Ethics Committee of the Tanzania National Institute of Medical Research (NIMR/HQ/R.8a/Vol.IX/3075) and the Advarra Review Board in the United States (Pr00034024). Written informed consent was obtained from all study participants.

## Results

### CHW and Malezi Program characteristics

Almost all (n = 107; 95%) of the enrolled CHWs completed the expected eight observations, with four observation records (two home and two clinic) at both baseline and endline. The CHW characteristics at baseline are presented in Table 1. CHW median age was 46 years (IQR 41, 52), almost two-thirds (63%) were female, most had been working as a CHW for eight or more years (IQR 6, 0), and almost one in five (19%) had received a secondary education. Information on the subset of CHW who participated in FGD can be found in supporting information (S3 Appendix).

**Table 1:** CHW characteristics at baseline.

	Baseline (n = 113)	
Assessed at baseline only	Median (IQR)	
Age (in years)	46	(41,5)
Years worked as CHW	8	(6,0)
	N (%)	
Sex		
Female	71	(63%)
Male	42	(37%)
Education		
Primary	91	(81%)
Secondary	22	(19%)
Where from		
In or near same community	63	(56%)
Other community in Tabora	12	(11%)
Outside of Tabora	38	(34%)
Number of children		
<3	11	(10%)
3–5	48	(42%)
6+	54	(48%)
Has under-5 child	24	(21%)
Holds community leadership role/position	26	(23%)

Baseline and endline characteristics for the CHWs and the Malezi Program are shown in Table 2. On average, CHWs reported working 4–6 days per month for about six hours per day, with each visit lasting around 30 minutes. These work effort estimates did not change from baseline to endline. However, estimations of work output, assessed by how often CHWs reported being able to conduct all their visits, increased from 46% at baseline to 62% at endline (p = 0.202). Using phones to make appointments (42% increasing to 83%) and to provide counseling services to caregivers (24% increasing to 49%) nearly doubled among the CHWs at endline (p < .0001). More than half (56%) of the CHWs reported spending “a lot of time” on early stimulation discussions with caregivers at endline, compared to 36% at baseline (p = .003). The majority of CHWs (71% at baseline, 81% at endline) also believed their work met community expectations (p = 0.071). Lastly, CHWs at endline reported high rates of video use (98% showed the videos more than 10 times in the past three months) and electronic sharing of the videos with caregivers (81%).

**Table 2:** CHW and Malezi Program characteristics at baseline and endline.

	Baseline (n=113)		Endline n=113)		p-value*
Assessed at baseline and endline	Median (IQR)				
Days worked/month	6	(4,12)	4	(4,8)	0.799
Hours worked/day	6	(4,8)	6	(4,7)	0.410
Confidence in ECD knowledge (7 item score; range 7-21)	19	(17,20)	20	(19,21)	<.0001
Satisfaction with CHW work (22 item score, range 0-88)	62	(55,68)	77	(69,82)	<.0001
Duration of visit in minutes	30	(20,30)	30	(20,45)	0.961
	N (%)				
CHW work output in past 3 months**					
Usually able to visit all assigned clients	52	(46%)	70	(62%)	
Sometimes or rarely able to visit all assigned clients	61	(54%)	43	(38%)	0.202
CHW made phone appointment for visits					
Usually or sometimes	48	(42%)	94	(83%)	
Rarely	65	(58%)	19	(17%)	<.0001
CHW provided service by phone					
Usually or sometimes	27	(24%)	55	(49%)	
Rarely	86	(76%)	58	(51%)	<.0001
CHW estimated spending “a lot of time” on early stimulation with pregnant women and caregivers of children under 5	41	(36%)	63	(56%)	0.003
CHW perceives their work meets expectations of community	80	(71%)	91	(81%)	0.071
CHW video use in past 3 months					
Shown to caregivers 10+ times			111	(98%)	
Shared with others electronically (SD card)			91	(81%)	

* Wilcoxon rank sum test used for continuous variables; McNemar’s test for categorical variables.

** Work output defined as a composite variable from two questions:

• Would you say you are usually, sometimes, or rarely able to visit every client on your list in the past three months? (usually=3; sometimes=2; rarely=1).

• About how many of your expected visits were you able to do in the past three months? (more than half or all=1; half or fewer=0).

### Planning and conducting home visits

During the FGDs, many CHWs reported making their own workplans. CHWs planned who was to be visited, the number of households to visit each day, visit timing/duration, and topics to be covered. CHWs covered a range of topics during the home visits, such as how to make toys/games, environmental safety, accessing the health facility, child stimulation, hygienic practices, family planning, and nutrition. A number of CHWs said they covered environmental safety or reviewed the reproductive health clinic card at every visit.

*“I must prepare what to teach before I leave home*. *That today I will go teach this and this*. *If it is on early child development, I will plan how to interact with a child*. *How to start? I will start with few games then move to the relevant topic*. *I will teach and teach and teach*. *After that, I will ask her to take me through how she relates with her child*. *And talk about hygiene and the safety of the child. After this, I will then talk about other topics partially, for example family planning among others*.*” (CHW)*

However, about half of CHWs described that plans had to be flexible to adapt to different situations in the household (e.g., urgent safety issues, child/family needs, presence of certain family members).

*“Sometimes, you can prepare yourself that you are going to teach ‘talking with the child’*. *But when you arrive in the family and find the child sick*. *You can’t start teaching them to talk with the child*. *You are supposed to encourage caregiver to take the child at a health facility*.*” (CHW)*

### Improvement of CHW counselling skills

The results of the CHW observations for clinic and home sessions are shown in Table 3. Overall, in the six dimensions (introduce, educate, ask, plan/problem solve, interact/encourage, and responsive care), the two dimensions of educate and ask decreased or remained the same. For example, at endline in the educate dimension, fewer CHWs scored well in educating caregivers about age-appropriate play activities or explaining the importance of play. Additionally, in the ask category at endline, fewer CHWs were able to ask initial questions about how the caregiver plays or communicates with the child as indicated in the counselling checklist. While the responsive care dimension increased, for home sessions it remained low. The remaining dimensions (introduce, plan/problem solve, and interact/encourage) increased significantly between baseline and endline in either clinic, home, or both settings.

**Table 3:** Quality of CHW counseling/mentoring: Performance of individual items common to the clinic and home tools, and used in final scoring and principal components analysis.

				Clinic			Home		
Dimension	Item		Response	Baseline (n=220)	Endline (n=224)	p-value	Baseline (n=222)	Endline (n=225)	p-value
				N (%)	N (%)		N (%)	N (%)	
Introduce	1.1	Explains the reasons for group session and what information will be discussed	Done	197 (89.5)	222 (99.1)	<.0001	182 (82.0)	222 (98.2)	<.0001
	1.2	Encourages caregivers to talk and ask questions at least once throughout the session	Done	46 (20.9)	120 (53.6)	<.0001	34 (15.3)	148 (65.5)	<.0001
	1.3	…the toys they are using in household	Done	132 (60.0)	158 (70.5)	0.020	112 (50.5)	140 (61.9)	0.014
Educate	2.1	Suggests play activities appropriate to at least two different child age groups	Done partly	15 (6.8)	27 (12.1)		24 (10.8)	39 (17.3)	<.0001
			Done well	190 (86.4)	163 (72.8)	0.002	163 (73.4)	124 (54.9)	
	2.2	Suggests communication activities appropriate to >2 different child age groups	Done partly	24 (10.9)	15 (6.7)		19 (8.6)	24 (10.6)	
			Done well	134 (60.9)	130 (58.0)	0.125	88 (39.6)	109 (48.2)	0.078
	2.3	Models stimulation behaviors or any best practices with at >1 child/caregiver	Done partly	23 (10.5)	22 (9.8)		43 (19.4)	43 (19.0)	
			Done well	140 (63.6)	137 (61.2)	0.76	98 (44.1)	102 (45.1)	0.98
	2.4	Provides clear explanation as to why play/communication activities are important to child development	Done partly	25 (11.4)	47 (21.0)		37 (16.7)	46 (20.4)	
			Done well	154 (70.0)	123 (54.9)	0.003	144 (64.9)	93 (41.2)	<.0001
	2.5	Provide examples of household toys appropriate to one or more age groups	Done partly	19 (8.6)	35 (15.6)		27 (12.2)	27 (11.9)	
			Done well	176 (80.0)	152 (67.9)	0.012	146 (65.8)	119 (52.7)	0.006
Ask		Asks (or gets responses from) >1 caregiver about ….							
	3.1	…how they play with their children	Done	203 (92.3)	204 (91.1)	0.65	201 (90.5)	145 (64.2)	<.0001
	3.2	…how they communicate with their children	Done	172 (78.2)	167 (74.6)	0.37	171 (77.0)	118 (52.2)	<.0001
	3.3	…how they get their children to smile	Done	158 (71.8)	139 (62.1)	0.029	160 (72.1)	86 (38.1)	<.0001
Plan / problem solve	4.1	…plans to do any play/communication activities at home, using “when, where, with what, with whom” probing	Done partly	21 (9.5)	54 (24.1)		16 (7.2)	24 (10.6)	
			Done well	41 (18.6)	46 (20.5)	<.0001	30 (13.5)	37 (16.4)	0.27
	4.2	…how they will engage the father (or others in household) to interact with the child	Done partly	14 (6.4)	66 (29.5)	<.0001	7 (3.2)	55 (24.3)	<.0001
			Done well	47 (21.4)	110 (49.1)		13 (5.9)	90 (39.8)	
	4.3	…challenges they may face in carrying out play/communication activities or increasing positive father-child interactions	Done partly	4 (1.8)	37 (16.5)		1 (0.5)	20 (8.8)	
			Done well	4 (1.8)	2 (0.9)	<.0001	2 (0.9)	22 (9.7)	<.0001
	4.4	Gives caregivers regular feedback by summarizing their statements or restating how they feel	Done partly	41 (18.6)	22 (9.8)		53 (23.9)	35 (15.5)	
			Done well	70 (31.8)	92 (41.1)	0.013	36 (16.2)	86 (38.1)	<.0001
	4.5	Responds appropriately and thoughtfully to caregiver	Done partly	49 (22.3)	40 (17.9)		73 (32.9)	60 (26.5)	
		comments or questions	Done well	109 (49.5)	143 (63.8)	0.008	69 (31.1)	114 (50.4)	<.0001
Interact / encourage		Engages at least one caregiver in discussion about …							
	5.1	CHW laid mat for caregiver(s)/child(ren) to sit?	Done	189 (85.9)	221 (98.7)	<.0001	94 (42.3)	197 (87.6)	<.0001
	5.2	CHW selected age-appropriate toys for the session?	Done	43 (19.5)	170 (75.9)	<.0001	101 (45.5)	148 (65.8)	<.0001
	5.3	Gives time for caregivers to practice playing and communicating with their children	Done partly	17 (7.7)	41 (18.3)	<.0001	33 (14.9)	41 (18.1)	
			Done well	118 (53.6)	141 (62.9)		94 (42.3)	133 (58.8)	<.0001
	5.4	Gives praise or appreciation to caregivers for how the CHW observes their behaviors during the session, or for	Done partly	50 (22.7)	21 (9.4)		20 (9.0)	2 (0.9)	
		questions asked, or comments made	Done well	110 (50.0)	192 (85.7)	<.0001	118 (53.2)	196 (86.7)	<.0001
	5.5	Encourages caregivers to raise any issues regarding their child’s health or development during or after the session	Done partly	20 (9.1)	11 (4.9)	0.002	63 (28.4)	53 (23.5)	
			Done well	187 (85.0)	211 (94.2)		121 (54.5)	157 (69.5)	0.001
Responsive care	6.1	Asks caregivers to share how they think the child communicates what the child needs or wants	Done partly	19 (8.6)	11 (4.9)	0.136	12 (5.4)	17 (7.5)	
			Done well	32 (14.5)	44 (19.6)		16 (7.2)	38 (16.8)	0.004
	6.2	Selects at least one caregiver/child pair to discuss how she observed her child and responded to her child	Done partly	12 (5.5)	7 (3.1)	0.469	13 (5.9)	21 (9.3)	
			Done well	39 (17.7)	39 (17.3)		11 (5.0)	27 (11.9)	0.008

Observation scores on CHW counseling skills, presented in Table 4, significantly increased overall in both clinic (+22%, p < .0001) and home (+29%, p < .0001) visits. In both types, the dimensions of *introduce*, *plan/problem solve*, *and interact/encourage* were significantly improved from baseline to endline. The remaining two dimensions, *ask and educate*, stayed the same or decreased (especially in home sessions, where both dimensions decreased by -33% and -14%, respectively).

**Table 4:** Quality of CHW counseling/mentoring: Clinic and home observation scores by round and session type.

Raw scores (23 items) Standardized scores (0–1 scale)* Sub-scale scores (refer to Table 2 for details)	Baseline (n = 442)	Endline (n = 449)	% change	p-value**
	Median (IQR)			
	N = 220	N = 224		
Clinic raw score	13.0 (11.0,16.0)	15.8 (11.0, 18.5)	+22%	< .0001
Clinic standardized score	0.56 (0.48, 0.70)	0.68 (0.48, 0.80)		
Introduce	0.67 (0.33, 0.67)	0.67 (0.67, 1.00)	0%	< .0001
Educate	0.80 (0.60, 1.00)	0.80 (0.50, 1.00)	0%	0.091
Ask	1.00 (0.67, 1.00)	1.00 (0.67, 1.00)	0%	0.181
Plan/problem solve	0.30 (0.10, 0.40)	0.40 (0.30, 0.70)	+33%	< .0001
Interact/encourage	0.60 (0.50, 0.80)	1.00 (0.80, 1.00)	+67%	< .0001
Responsive care	0.00 (0.00, 0.50)	0.00 (0.00, 0.25)	0%	0.267
	N = 222	N = 225		
Home raw score	10.5 (8.5, 13.0)	13.5 (9.5, 16.0)	+29%	< .0001
Home standardized score	0.46 (0.37, 0.57)	0.59 (0.41, 0.70)		
Introduce	0.50 (0.33, 0.67)	0.67 (0.67, 1.00)	+34%	< .0001
Educate	0.70 (0.40, 0.90)	0.60 (0.30, 0.90)	-14%	0.033
Ask	1.00 (0.67, 1.00)	0.67 (0.0, 1.00)	-33%	< .0001
Plan/problem solve	0.20 (0.00, 0.30)	0.40 (0.20, 0.60)	+100%	< .0001
Interact/encourage	0.50 (0.40, 0.70)	0.80 (0.60, 1.00)	+60%	< .0001
Responsive care	0.00 (0.00, 0.00)	0.00 (0.00, 0.25)	0%	0.002

* Each item earned 0–1 points; overall and sub-scale scores standardized to a 0–1 scale to adjust for varying number of items in each sub-scale.

** Using Wilcoxon Rank—Sum test.

**Fig 1:**
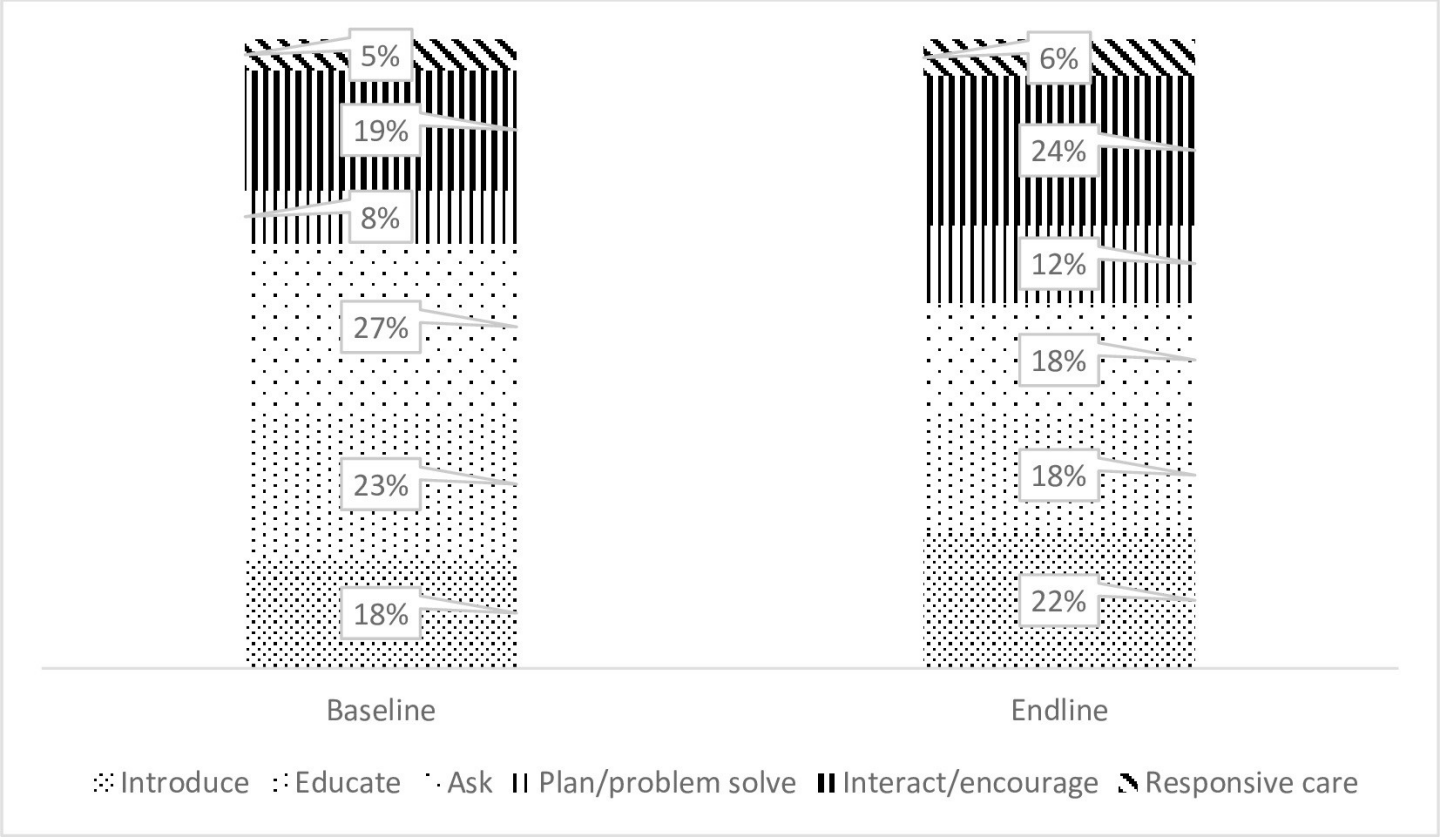
Contribution of CHW observation tool dimensions to total scores at baseline and endline. Video job aids enhance ECD messaging and promote ECD behaviors.

Results on the contribution of CHW observation tool dimensions to total scores (Fig 1) illustrated the significant shift in the counselling approach. Findings showed CHW skills moved away from the *educate and ask* approaches during baseline and towards *introduce*, *plan/problem solve*, *interact/encourage*, and *responsive care* at endline.

During counselling sessions, nearly all caregivers said CHWs would play the videos, sometimes pausing to emphasize points, explain the material and ask pointed questions. Many caregivers also said that the videos were supplemented with demonstrations of behaviors modeled on the videos, help them grasp and apply the information. About three-quarters of CHWs felt that the use of the ECD videos simplified their work, as they made concepts easier to explain and/or less challenging for caregivers to understand. A few CHWs further explained that the videos provided structure to the visit and better informed their counseling.

*“They did not understand well when we were using posters*. *They do understand better with videos because they see*. *They say we used not to understand when they said you should play…… now I see straight away when I watch the video*. *If I do these games*. *There are things that I am doing for the child, for instance, hugging the child*. *They narrate it*. *Therefore, the video is very important*.*” (CHW)*

For their part, the majority of caregivers reported practicing more ECD-focused behaviors after watching the videos. Most commonly reported was that they began playing or increased playtime with their child, followed by reports of increased communication between caregiver and child, implementation of more safety measures, and provision of more responsive caregiving. Over half of the caregivers stated they preferred videos be included with their home visits, while others did not have a preference. Among those who wanted videos used for all visits, their reasons included that the videos helped them understand the information better and more efficiently, they learned more from the visits that featured videos, and the videos showed them how to use ECD advice. However, the caregivers who were neutral reasoned that they learned from visits, whether or not videos were used.

*“I feel good because the video has made us learn a lot of things which we were not aware of*. *The video has taught us how to take care of and develop the children”*. *(Caregiver)*

*“I think they are both okay*. *When he comes and teaches just as we are without the video, we will understand*. *When he comes with a video, we will also understand*. *I don’t see any difference.” (Caregiver)*

While all caregivers stated that they liked the videos because they were educational and helped them to understand the ECD material, a few caregivers offered criticism. They reported being shown the same video at each visit, which they found boring and repetitive. This sentiment was echoed by one CHW.

### Home counseling sessions as opportunity for greater father involvement

The total number of caregivers attending clinic sessions decreased from about nine to five per session (p < .0001) and male participation in clinic sessions was virtually zero at both baseline and endline (Table 5). However, in home sessions, male participation significantly increased from 5.6% at baseline to 17.7% at endline (p < .0001), though the mother remained the primary caregiver present.

**Table 5:** Characteristics of clinic and home sessions and intervention fidelity measures.

	Baseline	During intervention	Endline	p-value*
Clinic sessions	Median (IQR; range)			
Number observed	N=232	n-344	N=226	--
Number female caregivers at group sessions	9 (7, 12; 4-27)	6 (4, 7; 3-24)	5 (4, 6; 3-12)	<.0001
Number male caregivers at group sessions	0 (0, 0; 0-4)	0 (0, 0; 0-3)	0 (0, 0; 0-2)	0.053
Duration of session in minutes	18 (13, 23; 5-77)	22 (18, 28; 5-88)	21 (18, 25; 5-40)	<.0001
Home sessions	N (%)			
Number observed	N=234	N=680	N=227	--
CHW visited household before	34 (14.7%)	623 (91.4%)	226 (100%)	<.0001
Who is present for the session (excluding index child <3 years)				
Mother	222 (95.7%)	646 (94.7%)	210 (92.5%)	0.42
Father	13 (5.6%)	118 (17.3%)	40 (17.7%)	<.0001
Co-caregiver (not mother or father)	16 (6.9%)	72 (10.6%)	30 (13.3)	0.076
Child(ren) 3-4	95 (40.9)	229 (33.6%)	70 (30.8%)	0.065
Child(ren) 5+	83 (35.8)	284 (41.6%)	95 (41.9%)	0.22
Duration of session in minutes	12 (8, 16; 5-72)	21 (17, 27; 6-97)	21 (16, 26; 6-47)	<.0001

In the IDIs with caregivers, all but one participant reported that their child’s father had participated in at least one home counseling session. During these visits, the CHWs engaged with fathers to teach them about and practice ECD-promoting activities; about two-thirds of caregivers reported that the father’s involvement with their child increased as a result. The most notable change was that more fathers were interacting and playing with their children, even when they had never done so before. Other changes in behavior included the father now comforting and calming his child, carrying and bathing his child, talking more softly when correcting a behavior or action, and making or buying toys.

*“Because sometimes, let me just say in the past, he didn’t use to play with his children*. *But when he saw its importance and how [the CHW] advised him, that’s when he started playing with the child”*. *(Caregiver)*

Several caregivers described CHW use of mobile videos to help educate fathers during home visits. Two caregivers also commented on how the ECD videos showcased fathers engaging with their children, not just the mothers. Similarly, some CHWs felt one of the advantages of the videos was that they emphasized and encouraged male participation.

*“Before, fathers didn’t know that they are also supposed to play with children but they are now aware, especially when they look at the videos and see other fathers playing with their children*. *They get motivated to also play with their children”*. *(CHW)*

However, a few CHW also acknowledged the difficulty in engaging fathers, which they felt was rooted in traditional gender roles (e.g., maternal caretaking role, father needing to be strict with children). A few caregivers indicated that the father’s level of engagement did not change after the CHW visits, but two of them noted this was because the father was already significantly involved in childrearing. Slightly fewer than half of caregivers were asked about the father’s limited or lack of involvement in CHW visits. Of this number, most respondents indicated that fathers were not home at the time of the CHW visits, due to work, travel, living elsewhere or an unexplained reason.

### Engagement with supervisor

Measures of CHWs’ supervisory engagement and appreciation increased over time, with findings presented in Table 6. At endline, more CHWs reported they had at least five one-on-one meetings with their supervisor in the previous three months (51% increasing to 75%; p < .0002). There were also increased rates of supervisors accompanying CHWs to the field for mentoring (60% increasing to 88%; p < .0001) and higher levels of supervisors providing feedback to the CHWs (81% increasing to 94%; p = .003). Overall, 42% of CHWs rated their supervisor as “excellent” at endline, compared to less than one in five (19%) rating them this way at baseline (p = .0002).

**Table 6:** CHWs’ supervision, mentorship, and relationship with their supervisors.

	Baseline (n=113)		Endline (n=113)		p-value*
Assessed at baseline and endline	Median (%)				
CHW met with supervisor one-on-one in the past three months					
5+ times	58	(51%)	85	(75%)	
<5 times	55	(49%)	28	(25%)	0.0002
CHW met with supervisor in a group in the past three months					
5+ times	98	(87%)	98	(87%)	
<5 times	15	(13%)	15	(13%)	1.00
Supervisor mentored CHW in field in the past three months	68	(60%)	100	(88%)	<.0001
Supervisor provided feedback in the past three months	91	(81%)	106	(94%)	0.003
CHW rated their supervisor as “excellent”	21	(19%)	48	(42%)	0.0002

* Wilcoxon rank sum test used for continuous variables; McNemar’s test for categorical variables.

## Discussion

This study evaluated the effect of ECD videos with mentorship and supervision to improve CHWs’ nurturing care/ECD counselling skills. Results showed changes in the types of counseling skills CHWs used and an overall increase in nurturing care counseling performance among the CHWs, as assessed through structured observations in the health facilities and during home visits. At baseline, CHWs were more likely to focus on asking and educating parents on nurturing care concepts, while at endline, a shift toward more structure and diversity of engagement approaches in counseling was observed. This included introducing the sessions, encouraging caregivers to interact with their children, problem-solving, and teaching responsive care practices. The study also noted improved supervision and mentorship from the CHW perspectives. Finally, findings supported the positive effect of the mobile videos on promoting ECD behaviors among caregivers and opportunities for father engagement in home settings.

One way the ECD mobile videos may have shifted and improved CHW counseling skills was that the videos helped them better structure sessions around specific talking points. This added structure from digital job aids, including videos, has been previously shown to improve workflow and the completeness and consistency of information [11]. CHWs also played the videos to model behaviors and then paused them to discuss, plan, and observe the caregivers practice those behaviors. This active use of the videos as a job aid might have helped CHWs to replace some of the more didactic elements of the counseling sessions, such as providing and gathering basic information, with softer and more caregiver-centered counseling skills. A systematic review of CHWs and mobile technology concluded that despite relatively low levels of education and minimal training among a community volunteer cadre, the use of video and other job aids could help to improve their performance in the provision of care [5]. In addition, the importance of using video demonstration to facilitate learning of new behaviors has been documented [19].

It is worth noting that engaging caregivers to discuss how they would undertake nurturing care activities provided an opportunity for self-reflection, problem solving, and the identification of individualized actions. A study on how CHWs used ECD videos in India found the tools helped them to better engage and educate clients [19]. Another study in India showed improved CHW performance through use of videos to engage women in dialogue to practice healthy behaviors [12]. Specifically, a parenting study in Jamaica, documented the benefit of CHWs using short films in health facilities to improve parenting practices [20].

Results from this study demonstrate the potential benefits of multi-pronged approaches to CHW capacity building and supervision. Where many projects may default to longer or more frequent in-service training sessions, deploying digital tools may add scaffolding that supports quality and consistency in service provision as well as opportunities for more structured supervision and mentorship after didactic training. Our study found CHWs reported an increase in the amount of mentorship visits where supervisors accompanied them to home visits, observing CHW performance and providing feedback. There were also more CHWs who rated their supervisor as “excellent” at endline. A positive and substantive relationship with a supervisor has been observed to be an important contributor towards CHW performance [5]. The importance of supportive supervision, including modeling and providing feedback to CHWs, was emphasized in a cluster randomized trial conducted in a parenting intervention in Jamaica [20]. In a literature review on CHW supervision in low-income countries, Hill et al [14] concluded that high-quality and frequent supervision visits improved CHW performance. However, a limitation of our study was that we did not directly measure supervisory/mentorship practices and the quality of supervision and mentorship likely varied according to the supervisor’s knowledge or commitment to working with CHWs. Thus, one area for further research is looking at how supervisors/mentors support CHWs to plan and conduct ECD-focused counseling.

Previous research has found that home visits by CHWs contribute to positive outcomes in relation to caregivers practices [21]. In our study, while still remaining relatively low, fathers’ involvement increased in the home environment, although no change was observed in the health facilities. Use of videos showing male participation in nurturing care practices may have been a contributing factor by demonstrating activities that fathers could replicate with their children. A similar study aiming to influence fathers’ behavior using videos modeling positive nutrition practices was conducted in Niger and showed positive results, such as spousal communication [22]. There is ample evidence that a father’s involvement in childrearing contributes positively towards short- and long-term outcomes of a child’s development related to health, mental, social, and emotional wellbeing [23]. Sharma et al [24] discussed ways to address the gap of male involvement in reproductive health services, which included improving knowledge and awareness on the topic to motivate male participation, creating male-friendly environments in health systems, and addressing social and cultural practices that inhibit male engagement, such as societal perceptions in participating in reproductive health services.

About 40% of our initially selected caregiver sample was replaced due to unavailability at the time of visit. It is possible that our study population was biased towards caregivers at home during the day. However, since we still reached a mix of caregivers in formal and informal employment, in rural and urban environments, and mothers who worked in and out of the home, we do not have reason to suspect there were major differences in characteristics that would have a significant impact on our findings. While we do not expect that environmental factors outside of the Malezi Program intervention led to the changes observed, it remains a possibility that the lack of a comparison group in this study has led to the over-estimation of the intervention’s effects. However, the group of participants included almost all available CHWs in the study area and used pre-post performance assessments of the same individuals to measure changes in counselling skills. In addition, there is the possibility of social desirability bias, such as expressing a positive view or displaying a behavior that the participant perceives the interviewer may want to hear; this could be amplified if the interviewers possess certain characteristics (e.g., older age, higher education level). Measuring how well the CHWs performed ECD counseling is difficult and the construct currently lacks a recognized and validated tool. A team of local ECD experts therefore adapted the UNICEF CCD counselling checklist and designed a program tool to mentor and supervise CHWs. This tool, which captured the different dimensions of ECD counseling, could make a useful contribution to ECD programs and future research.

Given the short timeline of project implementation and research, the sustainability of ECD-related services is uncertain. For example, newly recruited CHWs may not receive the full package of ECD training and supportive supervision through local government trainers once the project ends. In addition, the local government has minimal resources to procure new tablets or develop fresh digital content. However, the Malezi interventions were implemented through existing local government structures (health facilities, local government), and invested in training many facility and government staff in ECD and project-developed approaches to CHW supervision. In addition, the project worked closely with national leadership in developing guidelines and supporting policy reviews/adaptations from international recommendations.

## Conclusion

The study found the use of ECD videos, in combination with enhanced supervision and mentorship, improved CHW performance in providing counselling to caregivers in nurturing care practices. Recognizing the pivotal role CHWs can play in providing health-related services in under-resourced communities and the importance of home visits, it is essential to identify strategies that maintain the quality and consistency of CHW counseling to caregivers.

## Supporting information

S1 Appendix Malezi II CHW counseling checklist tool. (DOCX)

S2 Appendix Malezi II codebook with definitions and examples. (PDF)

S3 Appendix Community health worker (CHW) composition of focus group discussions (FGD). (DOCX)
