# Supplementary material for: Improvement of community health worker counseling skills through early childhood development (ECD) videos, supervision and mentorship: A mixed methods pre-post evaluation from Tanzania
Source: PLOS Glob Public Health. 2023 Jun 5;3(6):e0001152. doi: 10.1371/journal.pgph.0001152 (PMC10241410; doi:10.1371/journal.pgph.0001152)
Supplement: S1 Appendix — (DOCX) [file pgph.0001152.s001.docx]

**Effectiveness of Early Child Development Multi-Media Communication on Caregiver and Community Health Worker Behaviors: Evaluation of the Malezi II Program**

**Home-based Session CHW Observation Checklist**

| Village/area name: ______________________________ HH GPS: | |
| --- | --- |
| Facility catchment name (where CHW assigned): ______________________________ | |
| Session date:  **/** **/**  (*dd-mm-yy)* | Research Associate ID: |

1. **SESSION ELIGIBILITY SCREENING**

***Instructions to observer****: Answer the following questions based on your assessment of the home environment and caretaker/baby readiness for an observed session. Arrange to revisit another time if conditions make the session today ineligible, or select another household if the household appears ineligible.*

*A primary caregiver is someone who lives in the household, is recognized by others in the family/community as the child’s parent or guardian, and is responsible for the child’s eating and sleeping on most days of the week.*

1. Has the CHW visited this household before?

(1) Yes

(2) No 🡪 **ineligible HH**

1. Does any primary caregiver of a child under 3 years live in this household?

(1) Yes

(2) No 🡪 **ineligible HH**

1. Is any primary caregiver of a child under 3 years home now, with their child under 3?

(1) Yes

(2) No

1. Record the primary caregiver(s) and their child(ren) under 3 by age who will participate in the session. *Check all that apply.*

*(NOTE: The household is eligible with more than one caregiver and/or child under three)*

(1) Biological mother

(2) Biological father

(3) Grandmother

(4) Other, specify: ______________

1. Record details of all children under 3 present with the caregiver during the session

| **Sn.** | **age** | **sex** |
| --- | --- | --- |
| 1 |  |  |
| 2 |  |  |
| 3 |  |  |
| 4 |  |  |

1. Is any child under 3 awake, and expected to remain awake for the session?

(1) Yes

(2) No 🡪 **ineligible time, arrange to re-visit**

1. Is the selected caregiver able to attend and focus on the session for at least 30 minutes (not doing other work)?

(1) Yes

(2) No 🡪 **ineligible time, arrange to re-visit**

1. Is there a suitable place to have the session with regard to quiet, minimal interference/other observers, etc.?

(1) Yes

(2) No 🡪 **ineligible time, arrange to re-visit**

1. Indicate who is within sight and/or listening distance at the time of visit: *(select all that apply)*

(1) Co-caregiver (adult)

(2) Other (non-caregiver adult)

(3) Child(ren) 3-4 years

(4) Child(ren) 5+ years

***Based on responses to above screening questions, proceed with the CHW observation if eligible. Do not complete this checklist if household is ineligible. The session may be re-scheduled for observation, or another household may be selected.***

____________________________________________________________________

| Session start time:  : | Session end time :  : |
| --- | --- |

1. **CHW observations**

*Indicate yes, no or not applicable if indicated. Use the comment box as needed.*

|  | **Area preparation** | **Yes** | **No** | **Comments** |
| --- | --- | --- | --- | --- |
| 1 | Is there a mat laid for caregiver/child to sit? | Yes | No |  |
| 2 | Are toys organized on the mat according to age groups? | Yes | No |  |
| 3 | Is the caregiver/child sitting together and facing each other? | Yes | No |  |
|  | **CHW introduction** |  |  |  |
| 4 | CHW introduces self and greets caregiver/child at beginning of session | Yes | No |  |
| 5 | Explains the reasons for home session and what information will be discussed | Yes | No |  |
| 6 | Encourages caregiver to raise any issues regarding their child’s health | Yes | No |  |
|  | **Introductory counseling questions** | **Yes** | **No** | **Comments** |
| 7 | Asks caregiver about how s/he **plays with child** | Yes | No |  |
| 8 | Asks caregiver about how s/he **talks to child** | Yes | No |  |
| 9 | Asks caregiver about how s/he gets **child to smile** | Yes | No |  |
| 10 | Asks caregiver about the **toys used in household** | Yes | No |  |

*Indicate done well, done partially, not done, or not applicable if indicated. Use the comments box as needed.*

|  | **Counsel play/communication activities to strengthen caregiver/child interaction** | **Done well** | **Done partially** | **Not done** | **Comments** |
| --- | --- | --- | --- | --- | --- |
| 11 | Suggests **play activities** appropriate for at least one child age group | well | partly | not |  |
| 12 | Suggests **communication activities** appropriate to at least one child age group | well | partly | not |  |
| 13 | **Models stimulation behaviors** or any best practices | well | partly | not |  |
| 14 | Gives time for **caregiver to try out** new activities with his/her child | well | partly | not |  |
| 15 | Provides clear explanation as to **why play activities are important** to child development | well | partly | not |  |
| 16 | Provides **feedback on household toy** availability and age appropriateness | well | partly | not |  |
| 17 | Encourages **caregiver to observe what child does** and answer his/her cues | well | partly | not |  |
| 18 | Praise **caregiver** at least once during the session | well | partly | not |  |
|  | **Action plan and closing** | **Done well** | **Done partially** | **Not done** | **Comments** |
| 19 | Engages caregiver in discussion about **plans to do any play/communication activities** at home, using “when, where, with what, with whom” probing | well | partly | not |  |
| 20 | Engages caregiver in discussion about how they will **engage the father** (or others in household) to interact with the child | well | partly | not |  |
| 21 | Engages caregiver in discussion about **problems they may face** in carrying out play/communication activities or increasing positive father-child interactions | well | partly | not |  |
| 22 | Discusses how the **caregiver will solve the problems** (with play/communication activities or increasing positive father-child interactions) | well | partly | not  N/A |  |
|  | **General communication/observation skills** | **Done well** | **Done partially** | **Not done** | **Comments** |
| 23 | Gives caregiver regular feedback by summarizing their statements or restating how they feel | well | partly | not |  |
| 24 | Responds appropriately and thoughtfully to caregiver’s comments or questions | well | partly | not |  |
| 25 | Encourages caregiver to talk and ask questions at least twice throughout the session | well | partly | not |  |
| 26 | Faces body towards caregiver and child and maintains eye contact with whomever is speaking | well | partly | not |  |
| 27 | Was CHW sitting at the level of the caregiver/child most of the time? | mostly | partly | Not much / never |  |

|  | **Safety assessment** | **Done well** | **Done partially** | **Not done** | **Comments** |
| --- | --- | --- | --- | --- | --- |
| 28 | Mentions at least one potential or actual risk in the child’s environment (observed at current visit or as follow up from last visit) | well | partly | not |  |
| 29 | Discusses with caregiver at least one way to address risk or continue to minimize risk | well | partly | not |  |
|  | **Action plan and closing** | **Done well** | **Done partially** | **Not done** | **Comments** |
| 30 | Encourages caregiver to contact the CHW or health facility for issues that arise, or questions | well | partly | not |  |

1. **[ENDLINE ONLY] USE OF MOBILE VIDEOS**

|  | **Observer report** | **Response** | **Comments** |
| --- | --- | --- | --- |
| 31 | Did the CHW show any part of the ECD videos? | Yes  1  No  2 |  |
| 32 | If yes, which video(s) was/were played in full or in part?  *Check all that apply* | Video 1  1  Video 2  2  Video 3  3  Video 4  4  Video 5  5  N/A (no video shown today)  9 |  |

|  | **Use of mobile videos** | **Done well** | **Done partially** | **Not done** | **Comments** |
| --- | --- | --- | --- | --- | --- |
| 33 | Explains why these videos are being used | well | partly | not |  |
| 34 | Encourages caregiver to provide their thoughts and/or ask questions about things shown in the video | well | partly | not |  |
| 35 | Emphasizes and further describes key messages from the video | well | partly | not |  |
| 36 | Assesses caregiver’s understanding of messages from the video | well | partly | not |  |
| 37 | Pause the video during its’ showing to check understanding, discuss or demonstrate things from the video | well | partly | not |  |
