# Supplementary material for: Improvement of community health worker counseling skills through early childhood development (ECD) videos, supervision and mentorship: A mixed methods pre-post evaluation from Tanzania
Source: PLOS Glob Public Health. 2023 Jun 5;3(6):e0001152. doi: 10.1371/journal.pgph.0001152 (PMC10241410; doi:10.1371/journal.pgph.0001152)
Supplement: S2 Appendix — (PDF) [file pgph.0001152.s002.pdf]

Codes and Definitions Drafted: 9 December 2020

Malezi II Evaluation
Code List

THEMES, CODES & DEFINTIONS
THEMES are in ALL CAPS. Within each theme is a list of the codes that correspond to each theme. The themes are used as an organizing structure. Codes may be added as needed/they emerge from the data. Any code could be used with a CHW or caregiver. However, a few of the codes that mostly likely will only be addressed by CHW are starred with an *.

Code	Definition	Examples from transcripts
**A. CHW SUPPORT TO CAREGIVERS**		
A1a. CHWSupport:Positive/Neutral	These are **positive or neutral descriptions** by caregivers relating how they feel about the guidance received from CHW on parenting questions or challenges. This may include commentary on how knowledgeable they perceive CHW to be on these matters.<br><br>Also, use this to code **positive or neutral** caregivers' comments regarding CHWs' helpfulness in answering their questions or facilitating additional support.	R: My suggestions… (Laughs)… (Long silence). Me, how I see it, in my opinion, what I learn from the woman is much. About my child XXX…I have learnt a lot through this woman.
		**I: Please describe what do you mean when say [CHW] guidance is good?**<br>R: Because my child is sharp and grow up intelligently…They guide my child to use a toy, a ball and things which are not very sharp. I feel good…They motivate child's brain.<br>**I: And do you think what level of knowledge is/are CHWs have about these matters?**<br>R: They just have good knowledge.<br>**I: At what level?**<br>R: At the level of motivating children's Intelligence to be understood.<br>**I: And what makes you say they have good knowledge?**<br>R: They teach us and we understand.

Code	Definition	Examples from transcripts
A1b. CHWSupport:Negative	These are **negative descriptions** by caregivers relating how they feel about the guidance received from CHW on parenting questions or challenges. This may include commentary on how knowledgeable they perceive CHW to be on these matters (i.e., not very knowledgeable).<br><br>Also, use this to code **negative** caregivers' comments regarding CHWs' helpfulness in answering their questions or facilitating additional support	
A2. CHWSupport:CaregiversNeeds&Questions	Descriptions of health, development or social support **needs/questions raised** by caregivers to their CHW.<br><br>Also use this code when caregivers said they did not have any question to ask the CHW or did not have any needs.	**I: Okay. Remind me, what question did you ask her?**<br>R: I asked her a question about the child - "When the child is six months old, what is [the child] supposed to eat?" She told me that don't make the child of six months eat anything, even drinking water…only feed [the child] breast milk from the mother until [the child] reaches one year. That's when you feed [the child] porridge.
A3. CHWSupport:CommunicModality	This code is used to capture the **different ways caregivers may have reached out to a CHW with questions or support** (e.g., waited until CHW next home visit, phone call).	**I: Okay. So have you ever had a question, for example, you phoned her and asked "My child is feeling so and so, what should I do?"**<br>R: No, I have never phoned her.<br>**I: So, until she comes here, then if you have a question, you ask her.**<br>R: Yes.
A4. CHWSupport:ReasonsNotSoughtSupport	Responses offered for **why a caregiver did not ask for advice or support** for an issue related to their child. Responses may include felt CHW was too busy, need	**I: And you have not followed [CHW] looking for social support?**<br>R: I have not.<br>**I: Why you haven't?**<br>R: Just forgetting [while laughing]

Code	Definition	Examples from transcripts
	addressed by provider or someone else, did not think the CHW could help.	**I: How Forgetting?**<br>R: May be child has fever and I forget going to see [CHW]. I just go direct to the health facility
A5. CHWSupport:ExtentTrustCHW	The **extent** to which caregivers feel they can **trust CHW** and be open and honest with them.	**I: Okay. Personally, you feel you can trust, and be open and true to the CHW when she visits you here at home?**<br>R: Yes, I trust her. I trust her, and I ascertain that that woman is open…It's just according to how she is, how she teaches me, how she guides me…that's when I happen to trust and be open to her.
A6. CHWSupport:OtherInteractions	This code is for responses related to **places where caregivers meet CHW apart from homes visits and at the facility** and other details about that interaction. Responses that the caregiver has no interaction outside of home/facility can be coded here as well.	**I: What other places do you meet with her, apart from here at home when she comes to visit you, and when you take the child at the clinic?**<br>R: When she doesn't come here, we meet at the clinic. When I don't go, we don't meet anywhere, apart from her coming here to visit us.
		**: Are there any other places you meet with CHW apart from home visits and at the health facilities?**<br>R: Yes, At the health facility/ center.<br>**I: Any other place you meet [CHW]?**<br>R: I sometime go to his/her home…<br>**I: How do you interact with CHW when you visit at his/her home?**<br>R: I only go to discuss about children and showing me videos.
A7. CHWSupport:CHWLeadershipRoles	Any responses related to the **leadership roles** that CHW may serve in their communities can be coded here. No or 'don't know' responses can also be coded here.	**I: Okay. Is there any CHW you know who has leadership responsibilities, here in your society?**<br>R: Here? No. It's that woman only.
**B. HOME VISITS**		

Code	Definition	Examples from transcripts
B1. HomeVisits: Descriptions	Any **neutral description** of home visits, such as timing, frequency, etc, that are not part of recommendations or reflecting a positive or negative impression.	**I: And How many times is [CHW] coming?**<br>R: Three times per month.<br>**I: At the beginning you told me the last time you saw [CHW] was September?**<br>R: Was September, they came from Igunga.<br>**I: So they didn't come in October?**<br>R: They didn't come.<br>**I: Even in November, they didn't come?**<br>R: They didn't.
B2. HomeVisits: Planning	These responses should include descriptions of how CHW prepare for their household visits to meet the household's needs and address any issues. This can include a review of documentation from the last visit and any issues/questions that came up as well as how topics to cover (or to focus on) are determined for each session. Comments on topics that are always discussed or topics that there is often not enough time to discuss can be coded here.	
B3a. HomeVisitsImpressions:Positive/Neutral	Responses that reflect general **positive or neutral impressions of home visits** or the CHW conducting the home visits. If specifically related to the counseling or advice provided by CHW, these should be coded under 'positive perceptions of CHW guidance.'	**I: You have told me you feel good. Why do you feel good?**<br>R: I feel good because she comes to teach us how to be with children, how to parent the children – how we are supposed to care for them and what should we do for them for their mind growth…I feel good, because she teaches us many things, some of which we didn't know…now, she teaches us.
B3b. HomeVisitsImpressions:Negative	Responses that reflect **negative impressions** of home visits or the CHW	

Code	Definition	Examples from transcripts
	conducting the home visits. If specifically related to the counseling or advice provided by CHW, these should be coded under 'negative perceptions of CHW guidance.'	
B4. HomeVisits:Recommendations	Any **suggestions for how to improve the CHW home visit sessions**. Suggestions may include those related to timing, frequency, topics to cover, or other things caregivers would like to happen during home visits. Statements indicating no suggestions and/or that everything is okay/good/fine should also be coded here.	**I: And we are in December now, do you think they need to change number of visits? May be they should add reduce number of visits or how many times do you they have come?**<br>R: Just as current frequency.<br>**I: How many times per month?**<br>R: Three times.<br>**I: And when come here to teach, is there anything more about ECD and parenting topics you would like to learn but are not covered by CHW? Is there no anything you real prefer but it not covered by CHW?**<br>R: Nothing.
C. **FATHERS' INVOLVEMENT** – ***Please note that, the involvement of the fathers referred here is that when the father is actually present during CHW visit, and doesn't not include being involved by receiving education through the wife/female partner***.		
C1. Fathers'Involvement:How	This includes descriptions of **how** the CHW discusses early child development with the child's father, ~~the **importance** of these discussions~~ and the father's response.	**I: And do you think it is important to include father in those CHW home visits?**<br>R: Yes, if he is available it is important to include him.<br>**I: Why do you think it is important?**<br>R: We are all parenting our children with him.<br>I: **Any other reason, make you feel it important to include father as well.**<br>R: Because is our child,<br>**I: So How do the father contribute here?**<br>R: He just contribute good as well.<br>**I: In what ways?**<br>R: He also see importance of motivating children.

Code	Definition	Examples from transcripts
C2. Fathers'Involvement:Importance	This includes descriptions of caregivers thoughts on the **importance** of involving fathers in the ECD discussions.	**I: And do you think it is important to include father in those CHW home visits?**<br>R: Yes, if he is available it is important to include him.<br>**I: Why do you think it is important?**<br>R: We are all parenting our children with him.<br>I: **Any other reason, make you feel it important to include father as well.**<br>R: Because is our child,<br>**I: So How do the father contribute here?**<br>R: He just contribute good as well.<br>**I: In what ways?**<br>R: He also see importance of motivating children.
C3. Fathers'Involvement:WhyNotInvolved	This includes explanations of **why** the father **may not be involved** or may be unavailable during CHW visits (e.g., father does not live with the child and other caregiver).	
C4. Fathers'Involvement:Impact	This includes any examples or descriptions of how discussions between the father and CHW **resulted in any changes** in behavior, communication, household dynamics, etc. These changes can be positive or negative and directly or indirectly related to child development.	**I: Do you think are there any changes in the way father interact with a child after being included?**<br>R: Yes…A Child cries for his father when he is going way.<br>**I: Why child cries for his father now days?**<br>R: Because of parenting.
**D. OTHER ECD PLATFORMS**		
D1. OtherECDPlatforms:RadioSpots	Descriptions of radio spots about ECD that caregivers have heard. If a caregiver says that they have not heard any radio spots, their responses can also be coded here.	**I: So what were they saying what the advertisement was saying?**<br>R: [The neighbor] told me that the advertisement was explaining about children…to care for them.
		**I: You haven't heard any radio spot?**<br>R: I don't have a radio.

Code	Definition	Examples from transcripts
		**I: Even when you pass around Centre [village center] or at the milling machine, have not heard any radio spot about ECD and parenting playing out?**<br>R: I did not hear very well.
D2. OtherECDPlatforms:People&Places	This code includes **other places** in the community (besides home/facility) where child development and parenting knowledge should be delivered and **other groups of people (besides CHWs)** who should be providing information or counseling on early child development in communities. If anyone offers descriptions of where ECD is already being discussed/promoted, this can be coded here too.	**I: What other places do you feel that may be mothers usually assembly, for example, where if they receive the knowledge in those areas…or what other places when you personally go there, you receive knowledge of that kind? What other places?**<br>R: From what I know, this knowledge is given out from the medical facilities, or from those who visit us. Other places, no. On the side of the mothers, we ourselves go to learn there...
		**I: Apart from CHWs who else do think you can give out knowledge/counselling about Early childhood development and parenting?**<br>R: Government leaders.
D3. ECDPlatforms (CHW/radio):Behaviour Adoption	These should include responses regarding caregiver opinions about **whether or not people who** hear about ECD from CHWs or radio **will follow the advice/messaging and why**. Any characteristics or pattern of thinking of who is more or less likely to follow the advice can be coded here too (e.g., young/new parents, mothers, parents in rural areas)	**I: Okay. So, when people hear this message about early child development through the CHWs and also through radio spots, do you think they can follow that advice?**<br>R: There are others who follow…others don't follow it…There are others who forget about it…they say "these people are starting to teach us…we have searched for children by ourselves…someone else to come and start teaching us?" We don't follow…you find that we just forget about it.
		**I: Why the mothers?**<br>R: The mothers are the nurturers, yes? They should be listening, because the mothers are the nurturers of the children.
**PERCEPTIONS AND APPLICATIONS OF SHORT VIDEOS**		

Code	Definition	Examples from transcripts
**E. SHORT VIDEOS**		
E1. ShortVideo: Descriptions	This can include comments such as the number of different videos seen or if the same one has been used at each visit, descriptions of different videos they've seen (the one with the woman…, the one for young children, etc). Only include here standalone descriptions, that are not linked to positive or negative video perceptions nor to behaviors or things the caregiver has learned (use one of the three below).	**I: When she comes to show you the videos, are they different every time she comes, or she shows you the [same video]?**<br>R: It's just the same [video].<br>**I: And every time she comes, she shows you the same video again?**<br>R: It's just the same [video].
E2a. ShortVideoImpact: Play/Communication	Includes caregivers' descriptions of what they remembered/saw on the videos, what they have learned from the videos and how they have applied what they have learned to interactions with their child. Use this code for any examples related to **play or communication** activities. Can include responses about differences between child rearing before or after seeing videos (with the same children or different children).	R: What I have learnt…me as a mother…what I have learnt, me as a mother, from that short video…I have learnt to teach my child to greet, and to play like how he is playing…that's what I learnt.<br><br>**I: Three. For the senior ones, what had you never done to them, but after looking at those videos and learning from the CHW, you have started doing to this young one?**<br>R: To make him a car, to play with him like jumping with him…
E2b. ShortVideoImpact: ResponsiveCaregiving	Includes caregivers' descriptions of what they remembered/saw on the videos, what they have learned from the videos and how they have applied what they have learned to interactions with their child. Use this code for any examples related to **responsive caregiving** (e.g., getting child to smile, models behavior for child, observing/responding to child).	

Code	Definition	Examples from transcripts
	Can include responses about differences between child rearing before or after seeing videos (with the same children or different children).	
E2c. ShortVideoImpact:SafetyMeasures	Includes caregivers' descriptions of what they remembered/saw on the videos, what they have learned from the videos and how they have applied what they have learned to interactions with their child. Use this code for any examples related to **safety measures** (i.e., addressing risks in child's environment). Can include responses about differences between child rearing before or after seeing videos (with the same children or different children).	R: I saw bucket of water, holes and kerosene.<br>**I: What were they doing?**<br>R: We suppose to cover/close them, we should not leave them open.<br>**I: That is what you have learn?**<br>R: Yes…<br>**I: So after you have learned, how have you used those things to interact with your child.**<br>R: I make sure I cover the buckets and backfilling holes.
E2d. ShortVideoImpact:PositiveDiscipline	Includes caregivers' descriptions of what they remembered/saw on the videos, what they have learned from the videos and how they have applied what they have learned to interactions with their child. Use this code for any examples related to **Positive Discipline.** Can include responses about differences between child rearing before or after seeing videos (with the same children or different children).	
E3. ShortVideo:CaregiverLearningSupport	Descriptions of how did the CHW helped caregivers to learn from the videos. Responses may include stopping video at certain points to check understanding, providing additional demonstrations, or later reminding caregivers of information	R: She shows you…she stops [the video]…she asks you "Have you understood here? What did you understand?" Now you start explaining to her. After you watch, she stops it, she asks you "Mama XXX, have you understood here what they are teaching you?" If you haven't understood, you tell her. You tell her that "I haven't understood yet". She shows you again.

Code	Definition	Examples from transcripts
	from the video. If a caregiver says the CHW did not do any of these things, those responses should be coded here as well.	
E4a. ShortVideo:Posit/NeutraImpressions	Responses that reflect **positive or neutral impressions** of the use of videos during home visits. Responses that favor home sessions involving videos (or neutral or ambivalent responses) can be coded here. Comments may include the utility of seeing other families demonstrating behaviors or general satisfaction with them. These can include responses from caregivers or from CHW – on their own perceptions or on how they think caregivers perceive the videos.	**I: Do you think there was importance to you to see other families…because the video shows how another family does parenting…do you think it was important to you or good to you to learn from what others do in their nurturing?**<br>R: That is not very important. Even that of sitting and talking is important.
		**I: May be is it useful to see how other families interact which children in the video**?<br>R: Yes, there are playing good.<br>**I: And how do you find them, are you happy/enjoying to play them [videos]?**<br>R: Yes, am enjoying.<br>**I: Are you not thinking that, they are time wasting and makes home visits longer?**<br>R: They don't waste time.
E4b. ShortVideo:NegativeImpressions	This code includes responses that reflect **negative impressions** of the use of videos during home visits. Responses that favor home sessions without videos can be coded here. Comments may include that videos are unnecessary or waste of time that made the home visit longer, that they are repetitive, confusing, too long/too short, or general dissatisfaction with them. These can include responses from caregivers or from CHW – on their	

Code	Definition	Examples from transcripts
	own perceptions or on how they think caregivers perceive the videos.	
E5. ShortVideo/CHWs:OtherLessonsLearned	**Other things that caregivers have learned from the videos or from the CHW about early child development**, that are not captured in other codes.	
E6. ShortVideo:HomeVs.Facility	This code should capture differences in how CHW use mobile videos in the home versus in the health facility. Any differences in how they are received by caregivers in the different settings can also be coded here.	
E7. ShortVideo: AdvantageToCHW'sWork	These include descriptions of **how the videos have been helpful to CHW in their work.** Responses may include that the videos make it easier to show an example than explain or prompt good discussion/questions.	
E8. ShortVideo:Challenges	These include descriptions of how the videos have made CHWs' work more challenging. Responses may include that the videos disrupt sessions or take too much time.	
E9. ShortVideo:Recommendations	**Any suggestions for how to improve the videos.** Suggestions may include whole videos or segments of videos that should no longer be used or those related to timing (longer/shorter), more/different examples, or different actors. Statements	

Code	Definition	Examples from transcripts
	indicating no suggestions and/or that the videos are good as they are should also be coded here.	
**F. FACILITY ECD SERVICES**		
F0. FacilityECD:Descriptions	General descriptions of facility sessions. Only include here standalone descriptions, that are not linked to positive or negative perceptions of the seesions.	
F1a. GroupSessions:PositiveImpressions	This code includes any responses that reflect positive impressions of group sessions, what they like or enjoy about meeting in a group and receiving counseling/support through these sessions.	R: There, they usually sit – we are many…they sit, [and] start as if you are in class. We start being given knowledge, we are taught…The one with brains – the one who understands – will leave having understood…People who won't take the knowledge are also present…The one who will use the knowledge…
F1b. GroupSessions:NegativeImpressions	This code includes any responses that reflect negative impressions of group sessions, what they dislike about meeting in a group and receiving counseling/support through these sessions or any negative experiences they have had.	
F2. FacilityECD:CaregiversCHWInteractions	These include descriptions of **how caregivers and CHW engage at the health facility.** Examples of responses may be during individual or group	**I: Do you go at the health facility? And you find CHW? How is the interaction there, you're just greeting each other or [CHW] one giving the training/counseling?**

Code	Definition	Examples from transcripts
	counseling or more informal/casual/impromptu interactions.	R: Yes, we great each other the [CHW] start Counseling/training.<br>**I: Is [CHW] giving counseling `Individually or in group?**<br>R: Individually or in groups of two.<br>**I: Then you all get counselled in group?**<br>R: Yes<br>**I: The one doing so, Is CHW or nurse?**<br>R: CHW
F3. FacilityECD: CaregiversNeeds&Questions	Descriptions of health, development or social support **needs/questions** raised by caregivers to their provider.<br><br>Also, use this code when caregivers said they did not have any question to ask the facility provider or did not have any needs.	
F4. FacilityECD:ReasonsNotSoughtSupport	Responses offered for **why a caregiver did not ask for advice or support for an issue related to their child.** Responses may include felt provider was too busy, need addressed by CHW or someone else, did not think the provider could help.	**I: Now, let us leave the CHWs. Those medical providers - the nurses who you find at the clinic…have you ever asked any question or find assistance from a medical provider, meaning a doctor or a nurse, about the child's health or development of your child when you needed any help regarding your child?**<br>R: I have not done yet.<br>**I: The child has may be never got a disease, and you went to the hospital to get assistance from the nurse?**<br>R: Not yet.<br>**I: If it happens that may be the child has a cough, a fever, or whatever else, where from do you usually search for help?**<br>R: I usually go to the pharmacy. I carry him…I just explain…then he is given medicine.
F5. FacilityECD:ProviderHelpfulness	These are comments on the extent to which (none to large) caregivers felt their	

Code	Definition	Examples from transcripts
	**provider was helpful** in answering a question raised with them or facilitating additional support.	
F6. FacilityECD: Preference(Indiv.Vs.Group)	This includes caregiver preferences for learning about their child's early development and receiving support through individual counselling sessions with the provider versus group sessions. Reasons for their preference should be coded here too.	R: One by one is good, it's how we understand. In a group, you find that we argue against each other. But one by one, everyone leaves having understood.
F7. FacilityECD:GroupSessRecommendations	Any suggestions for how to improve the group counseling sessions at the health facility. Suggestions may include those related to timing, frequency, topics to cover, or more peer-to-peer interaction. Statements indicating no suggestions and/or that everything is okay/good/fine should also be coded here.	**I: How many time are they taking place currently**?<br>R: We usually do two times<br>**I: Two times per week?**<br>R: Yes<br>**I: How many time per week would you prefer**?<br>R: Same rate.<br>**I: Are there any other topics taught but are not covered there?**<br>R: No
F8. FacilityECD:Other	Use this for important segments of text that are not easily coded elsewhere. If there are multiple similar responses, a new code will be considered.	
**RELEVANCE OF THE MALEZI II PROGRAM**<br><br>**G. MALEZI II PROGRAM**		
G1. MaleziIIProgram:Posit/NeutrPerceptions	This includes any **positive or neutral thoughts or reactions that CHW described when they learned about the ECD program and their part in it.** Neutral	

Code	Definition	Examples from transcripts
	comments might include not knowing much or not having any expectations.	
G2. MaleziIIProgram:NegativePerceptions	This includes any negative thoughts or reactions that CHW described when they learned about the ECD program and their part in it. Any concerns that CHW had about the program at the beginning can be coded here as well, including a response that reflects no concerns.	
G3. MaleziIIProgram:CHWsExpectations	This code should capture CHWs' expectations for the ECD program and any comments on whether or not the program met or is meeting those expectations.	
G4. MaleziIIProgram:RelevanceCHWs'Work	These are comments related to the extent to which CHW feel the ECD program is relevant to their work as CHW and the reasons why they feel that way.<br><br>Use this code to also capture any impact of Malezi II program on CHWs work.	
G5. FacilityLevelChanges:Educat&Interact.	These include descriptions of how the provision of health education or interactions between CHW and caregivers at health facilities has changed since CHW were trained in the ECD program. Caregivers could also contribute responses here if they comment on any	

Code	Definition	Examples from transcripts
	changes they have noticed in care/services provided.	
G6. HouseholdLevelChanges: Educat&Interact	These include descriptions of how the provision of health education or interactions between CHW and caregivers in households/communities has changed since CHW were trained in the ECD program. Caregivers could also contribute responses here if they comment on any changes they have noticed in care/services provided.	
G7. MaleziIIProgram:Challenges*	These are barriers that CHW face in supporting caregivers on early child development. Responses may include not having enough time or resources or challenges related to confronting cultural norms.	
G8. MaleziIIProgram:HowAddressedChallenges*	These are strategies or approaches used by CHW to help address challenges faced when working with and counseling caregivers on ECD.	

***These will likely only be answered by CHW.**
