# Supplementary material for: Improvement of community health worker counseling skills through early childhood development (ECD) videos, supervision and mentorship: A mixed methods pre-post evaluation from Tanzania
Source: PLOS Glob Public Health. 2023 Jun 5;3(6):e0001152. doi: 10.1371/journal.pgph.0001152 (PMC10241410; doi:10.1371/journal.pgph.0001152)
Supplement: S3 Appendix — (DOCX) [file pgph.0001152.s003.docx]

**Supporting Information**

**S3 Appendix. Community health worker (CHW) composition of focus group discussions (FGD)**

|  | **FGD 1** | **FGD 2** | **FGD 3** | **FGD 4** | **Overall** |
| --- | --- | --- | --- | --- | --- |
| **District** | Igunga | Igunga | Nzega | Nzega | -- |
| **Number of CHW** | 7 | 8 | 8 | 6 | 29 |
| **Gender** | Male | Female | Male | Female | -- |
| **Age in years, median (IQR)** | 42 (27, 52) | 41 (43, 48) | 45 (39, 49) | 43 (41, 43) | 43 (41, 47) |
| **Performance level: mean combined clinic/home scores** | 14.5 | 13.8 | 13.2 | 13.7 | 13.8 |
